# Benthic Archives Reveal Recurrence and Dominance of Toxigenic Cyanobacteria in a Eutrophic Lake over the Last 220 Years

**DOI:** 10.3390/toxins9090271

**Published:** 2017-09-04

**Authors:** Benjamin Legrand, Amélie Lamarque, Marion Sabart, Delphine Latour

**Affiliations:** 1 Laboratoire Microorganismes Génome et Environnement (LMGE), Centre National de la Recherche Scientifique (CNRS), Université Clermont Auvergne, F-63000 Clermont-Ferrand, France; amelie.lamarque@uca.fr (A.L.); marion.sabart@uca.fr (M.S.); delphine.latour@uca.fr (D.L.); 2 ATHOS Environnement, 112 Avenue du Brézet, F-63100 Clermont-Ferrand, France

**Keywords:** sediment, Nostocales, akinetes, past blooms, *anaC* gene, anatoxin-a, *mcyA* gene, microcystin

## Abstract

Akinetes are resistant cells which have the ability to persist in sediment for several decades. We have investigated the temporal distribution of akinetes of two species, *Dolichospermum macrosporum* and *Dolichospermum flos-aquae*, in a sediment core sampled in Lake Aydat (France), which covers 220 years. The upper part, from 1907 to 2016, the number of akinetes fluctuated but stayed at high concentrations, especially for *D. macrosporum* in surface sediment (with the maximal value close to 6.10^5^ akinetes g DW^−1^ of sediment), suggesting a recurrence of blooms of this species which was probably closely related to anthropic eutrophication since the 1960s. Before 1907, the abundance of akinetes of both species was very low, suggesting only a modest presence of these cyanobacteria. In addition, the percentage of intact akinetes was different for each species, suggesting different ecological processes in the water column. This percentage also decreased with depth, revealing a reduction in germination potential over time. In addition, biosynthetic genes of anatoxin-a (*anaC*) and microcystin (*mcyA*) were detected. First results show a high occurrence of *mcyA* all down the core. In contrast, *anaC* gene was mostly detected in the surface sediment (since the 1980s), revealing a potentially more recent occurrence of this cyanotoxin in Lake Aydat which may be associated with the recurrence of blooms of *D. macrosporum* and thus with anthropic activities.

## 1. Introduction

Cyanobacterial blooms and the environmental nuisances they cause have increased continually over the last few decades due to anthropization of aquatic systems [1]. However, cyanobacteria are known to be one of the first photosynthetic organisms on earth [2] and their proliferations have occurred naturally for a very long time [3]. Thus, the real assessment of long-term cyanobacteria dynamics remains difficult. The sediment compartment, by stocking resting cells and macro-rests, represents a means of reconstituting the lake’s history; benthic archives are already known to store evidence of past aquatic ecosystems, including microbial diversity [4,5]. Concerning nostocalean cyanobacteria, their resistant cells, termed akinetes, have the ability to resist a huge panel of abiotic constraints, both in the water and in sediment, such as anoxia, desiccation and lack of light [6]. In this way, sediments contain real evidence of past nostocalean recurrences, and long-term data analysis make it possible to highlight the dynamics of past planktonic nostocalean diversity on a decennial time scale [7]. A few studies have already investigated past cyanobacterial recurrences in lacustrine ecosystems using fossil akinetes coupled with other markers [8,9] and have described nostocalean dynamics over 1950 years calibrated Before Present (cal. BP). However, no studies have considered the long-term viability of akinetes in sediment, which is used to assess the resistance of these cells. Only Wood et al. [10] tested the potential of the capacity of cells preserved in sediment to come back to life, and successfully detected akinete-forming nostocaleans in sediments dating back to 120 BP. The analysis of the 16S rRNA gene also helps to investigate the past distribution of cyanobacteria in sediment and cyanobacteria genes were detected over the last 150 years [10,11]. Parallel studies revealed the presence of potentially toxic cyanobacterial taxa, based on detection of the *mcyA* gene, throughout the last century in Lake Zurich [12]. The presence of *mcyB* mRNA in benthic populations of *Microcystis* buried in the sediment for the last few years has also been found in the Grangent reservoir (France) [13]. Nevertheless, most of these studies focus on the most ubiquitous hepatoxin, microcystin, even though a host of other cyanotoxins exist. Among them, anatoxin-a was one of the first cyanotoxins to be identified and characterized [14]. This toxin was particularly studied in lotic environments after dog poisoning [15]. Several studies from the last few years have also highlighted the presence of anatoxin-a in lacustrine environments, suggesting an unexpected occurrence [16,17,18]. Recent advances in molecular biology have allowed biosynthetic gene clusters to be characterized, and primers have been designed to highlight potential producers of anatoxin-a, including those present in the sediment [19].

To better understand patterns in species diversity and toxicity through time, it is essential to simultaneously integrate a quantitative characterization of past cyanobacterial proliferations, their past toxic potential and their capacity to survive long term in sediment. For this purpose, we used both microscopic and molecular approaches in order to reconstruct past nostocalean recurrences in terms of abundance, viability and toxic potential in a eutrophic lake (Lake Aydat, France). We identified the akinete distribution through a sediment core covering the last 220 years, and went on to characterize the physiologic state of akinetes in sediment, including the pool of intact akinetes. In parallel, we detected the presence of biosynthetic genes of two cyanotoxins, anatoxin-a and microcystin, first in total sediment and then focusing only on intact akinetes. Finally, we compared these patterns with other parameters such as watershed anthropization to examine the potential factors driving the long-term recurrence of Nostocales in Lake Aydat.

## 2. Results

### 2.1. Sedimentary Cored Chronology

**Figure 1:**
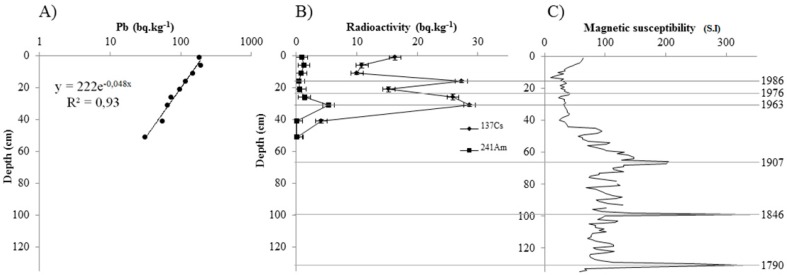
Chronology of the sediment core: (**A**) decrease of ^210^Pb; (**B**) peaks of ^137^Cs and ^241^Am; and (**C**) magnetic susceptibility.

Measurements of ^210^Pb provide an estimate of 0.65 cm year^−1^ for the mean sedimentation rate in Lake Aydat (Figure 1A) which is in accordance with the results of Lavrieux et al. [20] (0.6 cm year^−1^). The first peak of ^137^Cs (27.3 Bq kg^−1^) at 17.5 cm corresponds to the nuclear disaster of Tchernobyl in 1986 (Figure 1B). A second peak of ^137^Cs (28.6 Bq kg^−1^), combined with a peak of ^241^Am (5.37 Bq kg^−1^), is also observed at 32.5 cm. These correspond to the French nuclear assays performed in the Sahara desert in 1963. Measurements of magnetic susceptibility through the core can be divided into two major parts. The first (0–45 cm) possesses low values, including 0 and 45 S.I (Figure 1C), while values in the second part are higher at around 60–150 S.I. We have compared our profile with those of Lavrieux et al. [20], and the three major peaks that appear at 67, 99 and 131 cm correspond to major floods. These floods occurred in 1907, 1846 and 1790, respectively, according to Lavrieux et al. [20].

### 2.2. Vertical Akinete Distribution in Sediment

#### 2.2.1. Akinete Abundance along the Core

**Figure 2:**
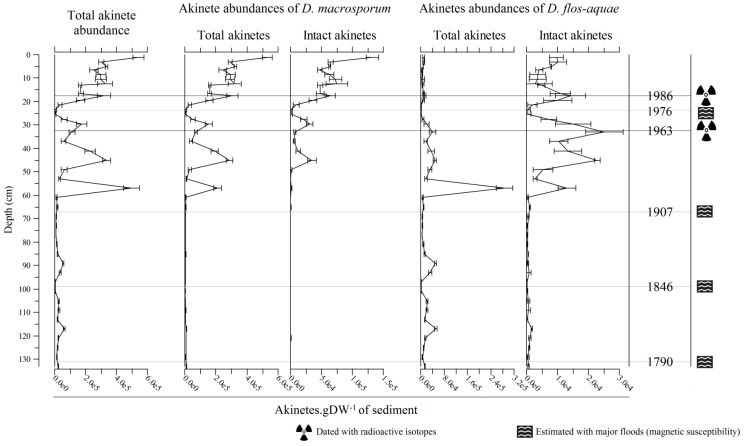
Distributions of total, *D. macrosporum* and *D. flos-aquae* akinetes through the sediment core.

Globally, the akinete distribution was negatively correlated with depth and magnetic susceptibility (Spearman correlation (SC) = −0.64 and −0.65 respectively, *p <* 0.001). Total akinete distribution along the core can be divided into three major parts (Figure 2): (1) the lowermost part, from 135 to 60 cm, was characterized by low akinete abundance, with between 3330 and 62,700 cells g of dried sediment^−1^ (g DW^−1^) of total akinetes with a mean value of 21,000 akinetes g DW^−1^ sediment; (2) the middle part, from 60 cm to 20 cm, was characterized by higher and very variable akinete abundance, with the number of total akinete between 490 and 498,000 cells akinetes g DW^−1^ sediment; and (3) the uppermost 20 cm were characterized by constant higher abundances of more than 150,000 akinetes g DW^−1^ sediment, which shoots up to 542,000 cells g DW^−1^ sediment at 1 cm. Two akinete morphotypes were found and counted along the core: ovoid and stick (corresponding, respectively, to *Dolichspermum macrosporum* and *Dolichospermum flos-aquae*). The vertical distribution of these two species was very different. *D. macrosporum* was more abundant and contributes mainly to the total akinete distribution, which shows a similar trend. For example, the correlation with depth was more acute with *D. macrosporum* (SC = −0.73, *p* = 4.16 × 10^−8^) than *D. flos-aquae* (SC = 0.28, *p* = 0.07). Total akinete abundance of *D. macrosporum* reached a maximum of 531,000 akinetes g DW^−1^ sediment in the surface sediment and then rapidly fluctuated up to 60 cm, with values close to 0 at 25.5 and 53 cm, and reaching 286,000 akinetes g DW^−1^ sediment at 45 cm. On the contrary, total akinete abundance of *D. flos-aquae* remained low and constant all along the core, with values of between 419 and 48,700 akinetes g DW^−1^ of sediment. The only anomalously high value came at a depth of 57 cm, with value of 288,000 akinetes g DW^−1^ of sediment. The akinete dominance between the two observed species evolved overall along the core (Figure S1). First, from the bottom up to 81 cm, *D. flos-aquae* was almost always the dominant species with more than 60% akinetes present in the sediment. This increased to 91% and 94% at 93 cm and 89 cm, respectively. Only twice during this sequence was *D. macrosporum* the dominant species, at 97 and 101 cm. Then, from 81 to 49 cm, the akinete distribution more or less equilibrated, with a slight dominance of *D. flos-aquae* at around 60% of total akinetes. Finally, the uppermost part (45–0 cm) was defined by the dominance of *D. macrosporum*, which was extremely marked in the top 25 cm where it exceeds 90%.

#### 2.2.2. Akinete Integrity along the Core

**Figure 3:**
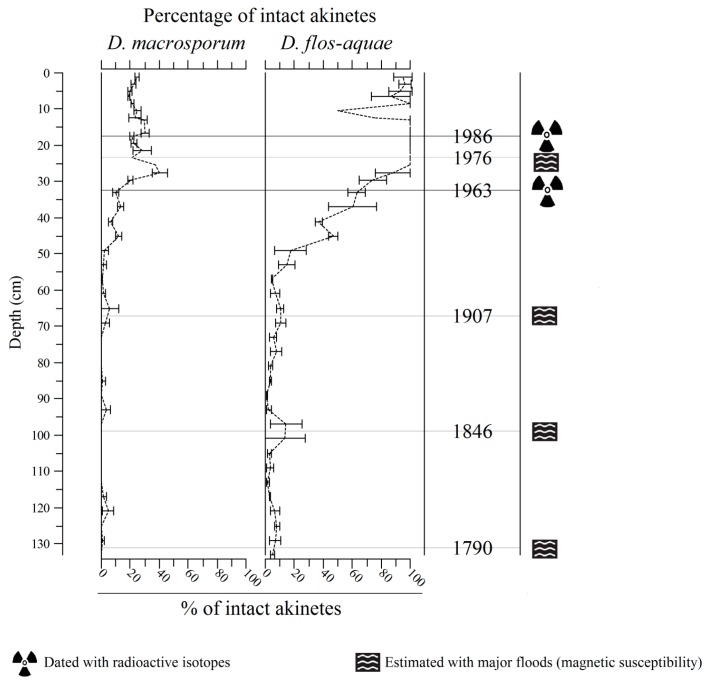
Percentage of intact akinetes of *D. macrosporum* and *D. flos-aquae* through the sediment core.

The percentage of intact akinetes fluctuates along the core (Figure 3) and was dependent on the species, especially in the upper part. From 29.5 to 0 cm, the percentage of intact akinetes was between 19% and 40% for *D. macrosporum*, whereas it was between 50% and 100% for *D. flos-aquae*, with mean values of 25% and 91%, respectively. Independent of this difference in percentage, the two kinetics along the core followed the same pattern (SC = 0.85, *p* = 7.3 × 10^−13^). This pattern is strongly negatively correlated with depth (SC = −0.84, *p* < 2 × 10^−12^), characterized by higher values and viability in the upper part. From 30 to 60 cm, the integrity progressively decreased to values of less than 10%. From this depth down to the bottom of the core, the integrity remained low, with values of between 0% and 5% and 1% and 14% for *D. macrosporum* and *D. flos-aquae*, respectively.

### 2.3. Target Gene Detection in Total Sediment

**Figure 4:**
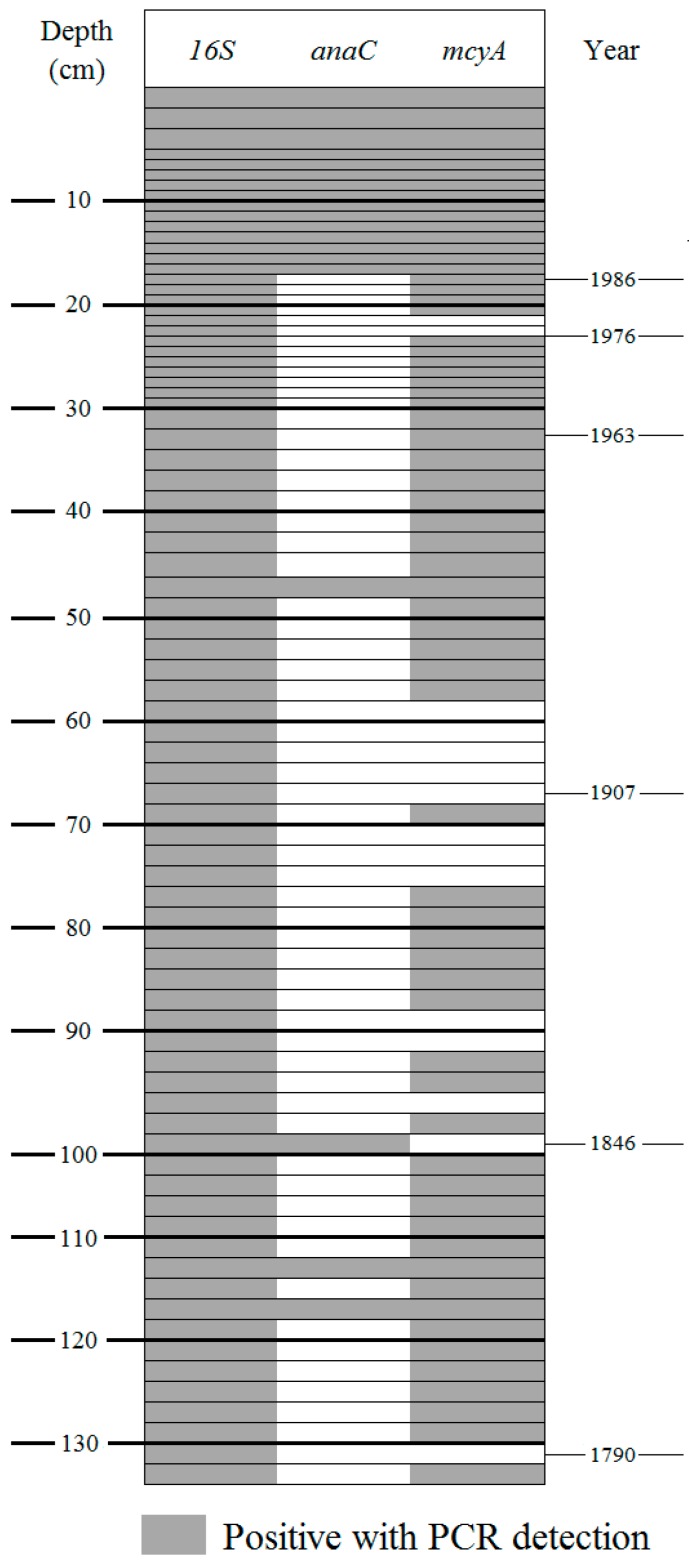
Detection of the targeted genes in total sediment. Layers represent the sampling performed along the sediment core (79 samples).

In total sediment, 16S rRNA gene was detected in all the studied samples (Figure 4). The *mcyA* gene, detected with classic PCR, is well represented and was detected in 64 samples. It was only absent from three major zones: zones 21–23 cm, 58–72 cm and 88–90 cm. In addition, this gene was also not detected in a few isolated samples (74–76, 98–100, 102–104 and 130–132 cm). *anaC* gene, detected using nested PCR, was found in the uppermost fourteen samples, corresponding to depths of 0–17 cm, which therefore included the same two samples detected with classic PCR (14–16 cm, data not shown). Nested PCR was also able to detect *anaC* genes in four other samples located at greater depth (46–48, 100–102, 112–114, and 116–118 cm).

### 2.4. Target Gene Detection in Extracted Intact Akinetes

**Figure 5:**
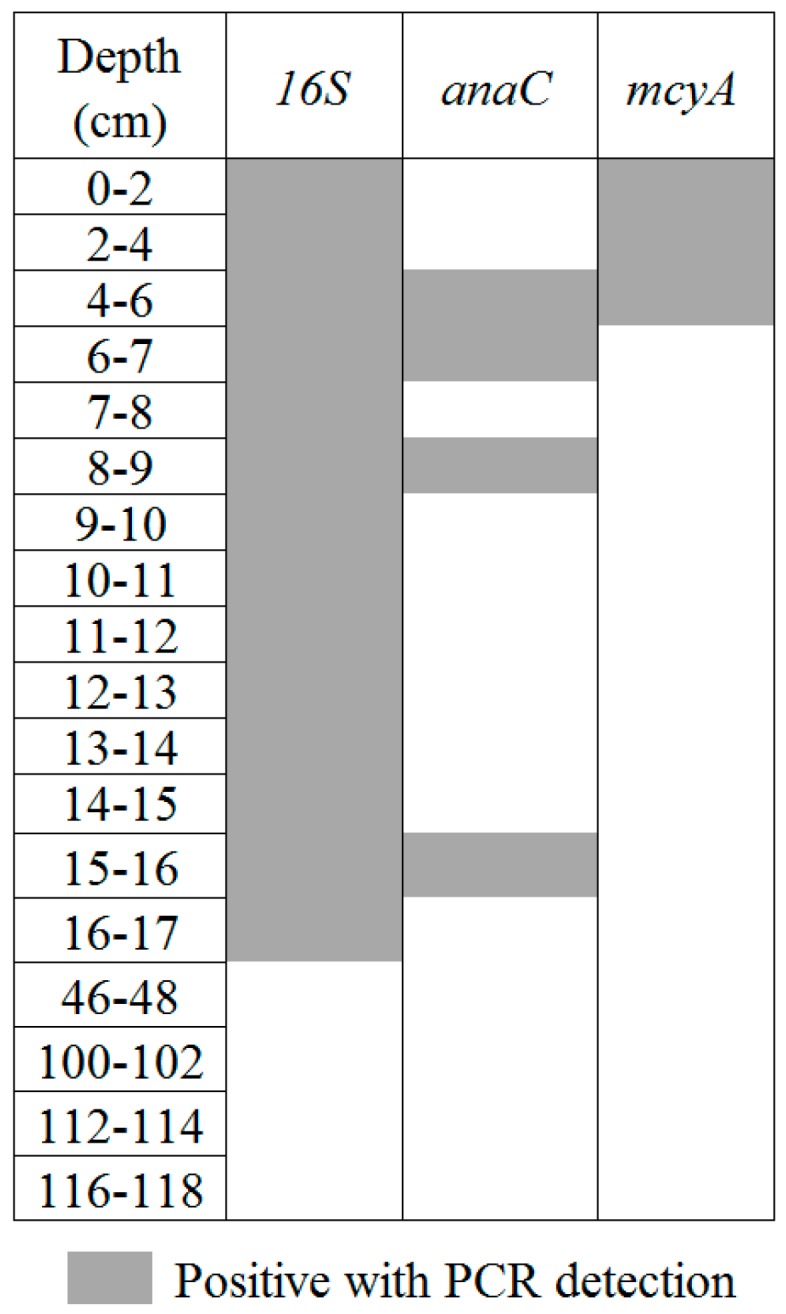
Detection of the targeted genes in intact akinetes.

The eighteen samples that have positive results in total sediment with both *mcyA* and *anaC* were chosen to look for these genes in the akinete cells. These samples correspond to the uppermost 14, from 0 to 17 cm, as well as segments at 46–48, 100–102, 112–114, and 116–118 cm (Figure 5). DNA extracted from intact akinete fractions revealed the presence of the 16S rRNA gene in all but the last four selected samples above 46 cm due to a low concentration of intact akinetes. The *mcyA* gene was detected in the three surface layers (0–2, 2–4, and 4–6 cm) and the *anaC* gene was detected in four samples (4–6, 6–7, 8–9, and 15–16 cm) exclusively with nested PCR.

### 2.5. Multiple Factor Analysis

**Figure 6:**
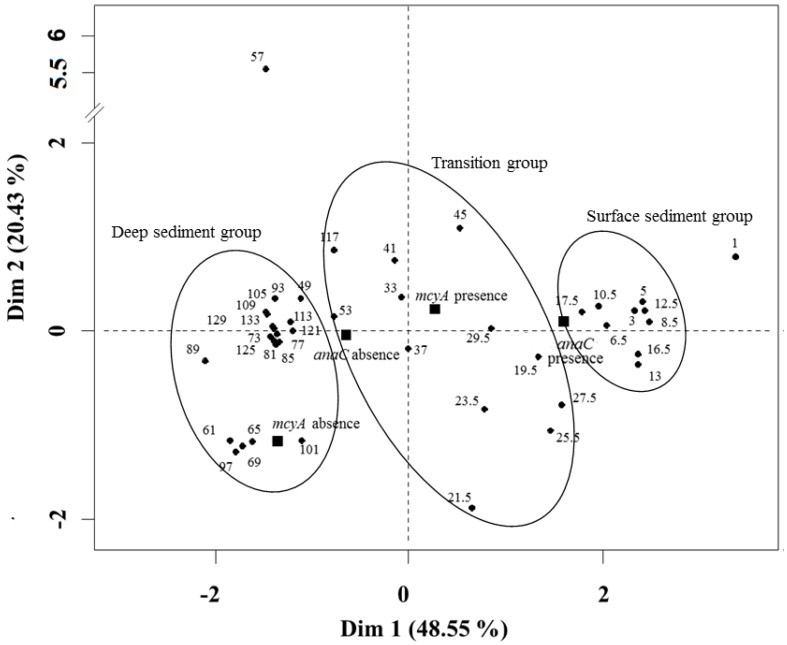
Individual factor map from the multiple factor analysis (MFA).

Multiple factor analysis including akinete abundances, akinete integrity and presence/absence of toxin genes allows samples to be divided into different groups (Figure 6). The first group is composed of surface samples (from 3 to 17.5 cm) characterized by *D.macrosporum* akinete abundance, high akinete integrity and the presence of *mcyA* and *anaC* genes (Surface sediment group). A second group is composed of all the samples from the bottom of the core and is characterized by low akinete abundance, low integrity and the absence of *anaC* genes (Deep sediment group). This group can be divided into two smaller groups depending on the presence or absence of *mcyA* gene. There is a progressive change between the surface sediment and the bottom sediment groups creating a transition group.

## 3. Discussion

We investigated the distribution of akinetes in a 220-year-old sediment core to reconstruct past nostocalean recurrences in Lake Aydat. This study highlights the presence of two species, *Dolichospermun macrosporum* and *D. flos-aquae*, which have already been detected in surface sediment of this lake [21]. Although akinetes of these two species were observed all along the core, drastic vertical changes are apparent, with high concentration in the recent part of the core, followed by low abundance in the oldest part. Two main hypotheses can explain this dynamic: (i) the disappearance of akinetes related to length of time spent in the sediment; and (ii) a past period, before 1907, with low nostocalean blooms. Lake Aydat is considered as having been eutrophic since approximately ca. 150 cal BP (around 1800 AD) [20]. Nevertheless, the 1800–1920 period was characterized as a period with strong erosion of the watershed [22]. This erosion, linked to the evolution of human practices within the watershed, and past climatic conditions (i.e., high rainfall inducing major floods but also the important detrital input from the Veyre River during this period) may have an effect on the water column stability and constitute unfavorable growth conditions for Cyanobacteria [23,24,25]. The negative correlation between total akinetes and magnetic susceptibility along the core (CS = −0.65 (*p* = 3 × 10^−6^)), supports this idea. Thus, the low abundance of akinetes in the deepest part of the core may be due to a period when there were no, or low blooms of Nostocales. On the other hand, the decrease of akinete integrity of both species in the deepest part of the core also suggests that akinetes may undergo deterioration with time. For example, akinetes in sediment may be damaged by physico-chemical processes such as pressure or sediment composition [9]. Unfortunately, there is no information, to our knowledge, about akinete survival rate in sediment. The only available information is related to the survival time in sediment: Livingstone and Jaworski [26] and Wood et al. [10] have highlighted that some akinetes may stay viable after 64 years and after 170 years, respectively. Furthermore, in our study, akinetes from around 110 years were also able to germinate (data not shown) despite a low percentage of intact akinetes. This indicates that even if the integrity of akinetes seems to rapidly decrease after a one hundred year duration in the sediment, some of them were still able to resist and re-germinate. This akinete resistance is probably driven at a species or genotype level, as the core evolved not only with time but also with species. In the uppermost 30 cm, *D. macropsorum* akinetes were characterized by a low integrity whereas a high one was measured for *D. flos-aquae*. The same applies in the surface sediment of Lake Aydat, where the percentage of intact akinetes varied from 7 to 60% for *D. macroporum* and *D. flos-aquae* respectively [27]. This high species-dependent variability may be explained by a species-specific sensitivity to ecological factors both in the water column and in sediment [21,27]. For example, it has already been highlighted that intact *D. macrosporum* akinetes were less resistant than intact *D. flos-aquae* akinetes for several abiotic factors such as desiccation [21]. Furthermore, this difference between species may be linked to parasitic interaction in the water column. *D. macrosporum* akinetes are known to be the specific host for the chytrid *Rhyzosiphon akinetum* [28]. For example, Gerphagnon et al. [29] have reported that 45.6% of the 92.3% *D. macrosporum* lysed akinetes present in surface sediment of Lake Aydat in 2014 were due to *R. akinatum* parasitism. Chytrid infection acts at a species or chemotype/genotype level, so akinete integrity can be affected at the same level and this could explain in part the observed difference in integrity between the two *Dolichospermum* species. Thus, in the core the akinete integrity dynamics may be the result of both the effect of time but also a fingerprint of the state of the akinetes in the water column. Even if an akinete had undergone cellular lysis, the empty wall may persist in sediment in the same way as diatom frustrules. For example, high abundances of *Aphanizomenon* fossil akinetes from 3900 years ago were detected in sediment from Lake Gosciaz (Poland) [8]. Akinetes are surrounded by a thickened cell wall and a multilayered extracellular envelope [30,31,32] composed of glucose-rich carbohydrate and amino compounds [33,34] which are able to persist in sediment. Thus, we suggest that, for our case, under a few hundred years, the total abundance of akinetes can give reliable information about past nostocalean blooms as already described in other studies.

After a period of low cyanobacterial abundance in Lake Aydat, the 1920–1960 period may be characterized as a transitional period between the lowermost and the recent sediment. This part is characterized by a succession of akinete peaks reflecting that the system was only weakly stable. Moreover, the only peak of *D. flos-aquae* in the entire core is in this section, which may confirm rapid fluctuations in the environmental conditions. This transitional period may also be linked to evolution of the ground cover in the watershed, as forest progressively recolonized the land, thus reducing erosion [22].

Finally, in the more recent part of the core, corresponding to the 1976–2016 period, the higher and stable akinete distributions highlighted a recurrence of nostocalean blooms. This is confirmed by the frequent observations of nostocalean blooms in the water column of Lake Aydat since at least the 1980s [35]. For example, Lafforgue et al. [36] reported a *Dolichospermum* bloom in autumn 1984. The marked dominance of *D. macrosporum* in the uppermost centimeters of the core seems to confirm the trend of monospecific blooms. During the 1960s anthropic development led to the progressive destruction of the wetland located just upstream of Lake Aydat and reduced the length of the Veyre River by almost 1 km [37]. This destruction of natural buffer areas, combined with agricultural practices, caused high nutrient concentrations in the Veyre River and then as input into the lake [38,39]. For example, total annual phosphorus input in the lake reached 640 kg P year^−1^ in the second half of the 1980s [36]. In the 1990s, this situation remained unchanged, with measurements of total phosphorus content carried out on the Veyre River upstream of Lake Aydat revealing high values of over 0.5 mg L^−1^ in June 1991 [34]. At the same time, the number of people living in the village of Aydat and in the lake’s watershed since 1975 has rocketed [40] probably creating a new input of nutrients into the lake. It is also now well known that an increase in temperature can be favorable to cyanobacterial blooms [24,41]. The 20th century has undergone two major phases of temperature increase: 1920–1945 and 1980–present [42]. Although it is very complicated to establish any direct link between global warming and blooms in Aydat, the increase in temperature during these periods may have enhanced the development of cyanobacteria.

This study also highlighted a past recurrence of the *anaC* gene in older sediment between the two oldest flood events of 1790 and 1846. To our knowledge, this is the first study that has detected this gene in deep sediment which has previously only been detected in the water column [17,18] or in surface sediment in lacustrine systems [21]. Nevertheless, it is surprising to find the *anaC* gene in only a few older samples, and then find a larger recurrence at the top of the core. This recent recurrence of the *anaC* gene may be associated with the recent dominance and omnipresence of *D. macrosporum* in Lake Aydat since the middle of the 1980s. Moreover, this gene was also detected in intact akinetes extracted from the sediment. We can therefore assume that *D. macroporum* possesses this gene and that the large increase in this species over the last 30 years has promoted the recurrence of *anaC* gene. This is the first time that a combination of microscopic and molecular techniques has been used to show up a long scale recurrence of a toxigenic cyanobacterium. On the other hand, the low number of positive samples of intact akinetes extracted from the sediment compared to positive samples of total sediment indicates that the target genes were mainly present as free-DNA in sediment particles, as suggested for surface sediments from ten French sites [19]. Furthermore, this weak percentage of toxic genotypes in intact akinetes suggests that a proportion of akinetes possessed biosynthetic genes and another part did not. This heterogeneity has already been highlighted in the water column for numerous toxigenic species [18,43,44], as well as in akinetes from surface sediment [21]. Thus, these genes may derive from *Dolichospermum* vegetative cells or lysed akinetes but can also come from other cyanobacterial genera from others orders.

The vertical distribution of the two studied genes of cyanotoxins is very different throughout the core. Concerning microcystin and the *mcyA* gene, the major recurrence of *mcyA* along the entire core indicates a significant occurrence of this toxic genotype over the last 220 years. The same pattern was also observed in Lake Zurich, linked to the presence of *Planktothrix rubescens* [12]. Even though this species has never been detected in Lake Aydat, numerous microcystin-producing genera have been found: *Microcystis*, *Woronichinia*, *Pseudanabaena* [18,36]. As some samples of the intact akinetes have positive detections of the *mcyA* gene, we can infer that at least one of the two studied species of *Dolichospermum* also possessed this gene. This is in accordance with the literature; some *Dolichospermum* are known to produce microcystin [45]. Thus, a mix of microcystin producers through time may be possible. In addition, detection of *anaC* and *mcyA* genes in some of the same samples suggests a past co-occurrence or at least of close succession of genotype producers of anatoxin-a and microcystin in the water column at Lake Aydat. The co-occurrence between microcystin and anatoxin-a has already been highlighted in the water column at different sites [46,47,48,49] well as at Lake Aydat in 2011 [18]. Contrary to these earlier studies, our results reveal that this co-occurrence is not recent but has been present since the middle of the 19th century, becoming recurrent from around 1986. However, even though the appearance and/or disappearance of toxic genotypes is clearly highlighted throughout the core, providing interesting information in terms of lake management [50], the possible mechanisms which triggered these phenomenon, such as those implied by annual variations in the water column [18,43,44] remain unexplained.

## 4. Conclusions

We have used a combination of microscopic and molecular tools to determine the past distribution of *D. macrosporum* and *D. flos-aquae* in Lake Aydat over the last 220 years. They showed that, after a period of low cyanobacterial abundance, probably linked to strong erosion within the watershed, a marked recurrence of *D. macrosporum* occurred from at least the 1980s, probably closely related to human interference. From the latter period to the present day, *anaC* gene became omnipresent, inducing a major co-occurrence of *mcyA*. These cellular and molecular patterns may suggest that anthropic pressures, in addition to promoting the increase of the *D. macrosporum* biomass, can also lead to the recurrence of a toxic genotype. New integrated studies, including akinete distribution and detection of cyanotoxin genes, need to be performed over long time scales (several thousands of years) to have a better understanding of the mechanisms (i.e., climatic changes or human intervention) that trigger cyanobacterial proliferations.

## 5. Materials and Methods

### 5.1. Study Site and Sampling

**Figure 7:**
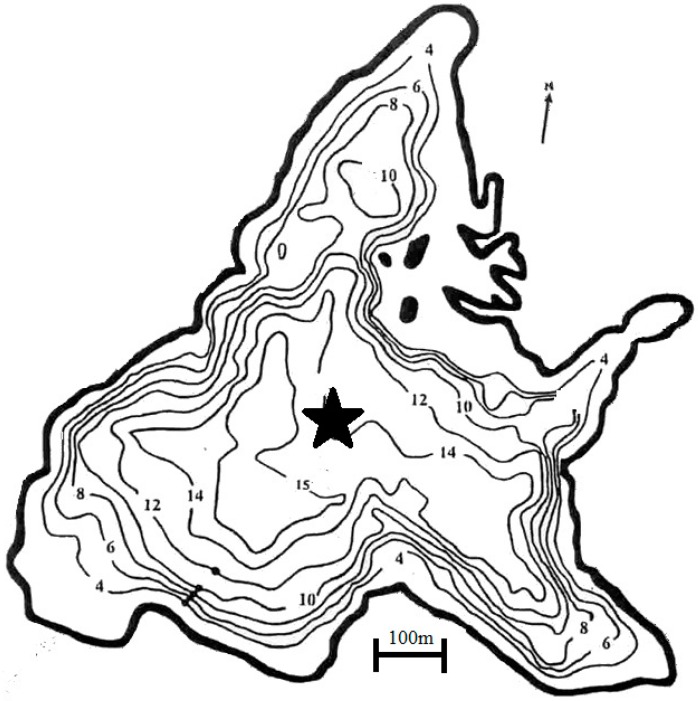
Bathymetric map of Lake Aydat. Star represents the point where the core has sampled.

Lake Aydat is located in the French Massif Central (45°39′48.9′′ N; 2°59′07.8′′ E) and is 837 m above sea level. It is a natural lake which was formed when the Veyre River was dammed by a basaltic lava flow 7500 years ago [20]. It is a small dimictic lake with a total area of 60 hectares, a large catchment area of 30,000 hectares and a maximal depth of 15 m. It is a eutrophic lake with recurrent cyanobacterial proliferations, especially of *Dolichospermum macrosporum* in autumn. These cyanobacterial proliferations led to a ban on nautical activities (including swimming, sailing and fish consumption) in September 2009 and again in August 2017. A sediment core of 1.35 m was obtained at the deepest point (Figure 7) with a sediment corer (UWITEC devices, Mondsee, Austria). The core was stored in dark conditions at 4 °C prior to analysis.

### 5.2. Core Characterization, Sampling and Dating

The core was sliced into 79 sample slices of 1 to 2 cm thick depending on the stratification. To obtain a dry weight, 1 g of fresh sediment of each sample was dried at 60 °C for five days. Dating of the sediment was carried out using three radioactive isotopes naturally present in this environment: ^137^Cs, and ^241^Am and ^210^Pb. The use of ^210^Pb originating from the decay of atmospheric ^222^Rn is a well-established method to estimate sedimentation rate [51]. ^137^Cs and ^241^Am are artificial radioisotopes with respective half-lives of 30 and 432 years. The radioisotope ^241^Am is observed only in weapons testing fallout up to 1963, when the Partial Test Ban Treaty was signed; however, ^137^Cs is present in fallout prior to 1963, as well as in later events such as the Chernobyl disaster in 1986. It is thus possible, by simultaneously measuring these two radionuclides, to date the peak of ^137^Cs in a core: 1963 corresponds to peaks of ^137^Cs and ^241^Am, whereas 1986 corresponds to a ^137^Cs increase alone [52]. The sediment was first dried at 110 °C for 24 h. High-efficiency gamma-spectrometry was measured at the “Laboratoire Souterrain de Modane” as described in Reyss et al. [53]. The results are expressed in units of Bq kg^−1^. In addition, magnetic susceptibility was measured following a step of 1 cm using a Bartington MS2E point sensor on a Multi-Sensor Core Logger System. Moreover, magnetic susceptibility measurements can be used as markers of detrital input from the Veyre. This method can thus highlight brief events such as floods and other much longer-term changes such as variations in the erosion flux due to global climatic conditions or human watershed occupation [20].

### 5.3. Akinete Extraction from Sediment and Microscopic Counts

The akinetes were separated from the other organic and mineral particles using a density gradient already described in Legrand et al. [21]. Briefly, 0.5 g of fresh sediment was diluted with 9.5 mL of distilled water and 4 mL of ludox TM 50 (Sigma-Aldrich, Saint Louis, MO, USA). Then, samples underwent sonication (30 s, frequency: 50%, power: 80 W, Sonoplus Bandelin^®^) and centrifuging (10,000 G for 30 min at 4 °C) steps. Finally, 4 mL of ludox supernatant containing akinetes was pipetted, homogenized, and then aliquoted with a final volume of 2 mL. For each sample, two extractions were performed, thus four replicates were counted. Akinetes were enumerated and discriminated with morphological criteria [21,27,54,55]. Moreover, this determination was completed by analyzing the morphology of young filaments on germinating akinetes (data not shown) using taxonomic keys in reference books [56,57,58].

To discriminate intact akinetes from empty ones, 2 μL of SYTOX GREEN^®^ (Invitrogen, Carlsbad, CA, USA) (50 μM) was added in each aliquot following Legrand et al. and Gerphagnon et al. [21,59]. Solutions were incubated for 30 min at 4 °C in the dark. These were then filtered on 8 μM filter (TEPT filters, Merck Milipore, Tullagreen, Ireland) under pressure (around 53 kPa). For each replicate, forty fields were counted with an epifluorescence microscope with a biomagnification of 160× (Zeiss Axiovert 200 M). For each field, two counts were performed: the first at 546 nm to enumerate all akinetes using the autofluorescence of chlorophyll pigments; and the second at a light emission of 488 nm to discriminate damaged akinetes [21].

### 5.4. Akinete Purifications

Akinetes from the sediment were purified in order to extract DNA only from intact akinetes with a protocol modified from Legrand et al. [21]. Akinetes from 1 g of fresh sediment were extracted following the ludox protocol described above. The akinete-containing solution was first cleaned through a 100 μm and then a 50 μm nylon tissue in order to eliminate larger particles and phytoplankton/cyanobacteria colonies. Then the supernatant was filtered through a 10 μm nylon tissue which was cleaned with a minimal volume of distilled water. Filtrates were pelleted by centrifuging at 10,000 G for 10 min at 4 °C. Supernatants were thrown away and each pellet was suspended in 600 μL of molecular water. Then, samples were treated with DNase (DNA-free kit, Thermo Fisher Scientific, Waltham, MA, USA) following manufacturer instructions. A cleaning step was performed by centrifuging (10,000 G for 10 min at 4 °C). Pellets were suspended in 600 μL of molecular water. This step was repeated three times and the three supernatants for each sample were kept and analyzed in the same PCR conditions as the akinetes samples in order to check there was no residue of free-DNA present. Finally, 100 μL of this solution was used to check for the absence of cyanobacterial colonies or vegetative cells and 500 μL was used for extraction of DNA from akinetes.

### 5.5. DNA Extractions from Total Sediment and Purified Akinetes and Cyanobacterial 16S RNAr Gene Amplification

DNA from total sediment and from purified akinetes was extracted using a FastDNA^®^ Spin kit for soil (MP biomedicals^®^), following the manufacturer’s instructions. Approximately 0.5 g of sediment and 500 μL of purified akinete solution were used to perform each extraction. To test for the presence of cyanobacterial DNA and the absence of PCR inhibitions, 16S cyanobacterial gene was targeted for each sample with primers from Nubel et al. [60]. The PCR mixture was composed of 5 μL of colorless Go Taq^®^ felxi 5X Buffer, 2.5 mM of MgCl_2_ solution, 0.2 mM of dNTPs, 0.4 μM of cya359F and cya781R primers (Table 1), 1 mg mL^−1^ of Bovine Serum Albumin (BSA), and 1.5 U of GoTaq^®^ G3 Hot Start Polymerase in a final volume of 25 μL. The PCR program was the same as in Legrand et al. [19,21].

### 5.6. Cyanotoxin Gene Amplification

One biosynthetic gene of anatoxin-a (*anaC*) and one of microcystin (*mcyA*) gene were investigated in this study. *mcyA* detection was performed with classic PCR, whereas nested PCR was also used to detect *anaC* gene. *anaC* was targeted with anxgen-F2 and anxgen-R and anaCgen-F2-anxgen-R for nested PCR. *mcyA* genes were detected with mcy A-Cd 1F and mcy A-Cd 1R respectively (Table 1). PCR mixtures were the same as in Legrand et al., 2016b [19] for anxgen-F2 and anxgen-R and anaCgen-F2-anxgen-R. PCR programs used were referenced in Legrand et al. [19,21] for *anaC* and in Hisbergues et al. [61] for *mcyA*. For *mcyA* the mix was composed of 5 μL of colorless Go Taq^®^ felxi 5X Buffer, 4.5 mM of MgCl_2_ solution, 0.2 mM of dNTPs, 0.4 μM each primer (Table 1), 1 mg mL^−1^ of Bovine Serum Albumin (BSA), and 1.5 U of GoTaq^®^ G3 Hot Start Polymerase in a final volume of 25 μL. All PCR products were revealed with 0.5% agarose gel with 0.3 mg L^−1^ ethidium bromide and migrated in a TAE buffer 1× at 100 V for 30 min.

### 5.7. Statistical Analysis

Spearman correlations were performed on all studied parameters using the software version 3.04 (Øyvind Hammer, Natural History Museum, University of Oslo).

Multiple factor analysis (MFA) was performed with the R software, package Rcmdr, version 1.6-1. Two quantitative groups were chosen for this analysis: akinete abundance with two variables, *D. macrosporum* and *D. flos-aquae* akinete abundance; and akinete integrity with two variables, *D. macrosporum* and *D. flos-aque* integrity. The qualitative group was defined by the absence or presence of *mcyA* gene and *anaC* gene.

## Figures and Tables

**Table 1:** Primers used to detect targeted genes.

Type of PCR	Primer Name	Sequence (5′-3′)	Target Gene	Gene Amplicon Size (bp)	References
Classic	cya359F	GGGGAATYTTCCGCAATGGG	Cyanobacterial	403	[60]
PCR	cya781R	GACTACTGGGGTATCTAATCCCATT	16S rRNA gene		
Nested	anxgen-F2	ATGGTCAGAGGTTTTACAAG	*anaC*	861	[19] modified
PCR 1	anxgen-R	CGACTCTTAATCATGCGATC			from [17]
Nested	anaCgen-F2	TCTGGTATTCAGTMCCCTCYAT	*anaC*	519	[19] modified
PCR 2	anxgen-R	CGACTCTTAATCATGCGATC			from [17]
Classic	mcyA-Cd 1F	AAAATTAAAAGCCGTATCAAA	*mcyA*	291	[61]
PCR	mcyA-Cd 1R	AAAAGTGTTTTATTAGCGGCTCAT			

## Supplementary Materials

The following are available online at www.mdpi.com/2072-6651/9/9/271/s1, Figure S1: Relative percentage along the sediment core between total akinetes of *D. macrosporum* and *D. flos-aquae*.
