# Supplementary material for: Benthic Archives Reveal Recurrence and Dominance of Toxigenic Cyanobacteria in a Eutrophic Lake over the Last 220 Years
Source: Toxins (Basel). 2017 Sep 4;9(9):271. doi: 10.3390/toxins9090271 (PMC5618204; doi:10.3390/toxins9090271)
Supplement: Supplementary file 1 [file toxins-09-00271-s001.pdf]

*Toxins* **2017**, *9*, 271; doi:10.3390/toxins9090271 

# Supplementary Materials: Benthic Archives Reveal Recurrence and Dominance of Toxigenic Cyanobacteria in a Eutrophic Lake over the Last 220 Years

**Benjamin Legrand, Amélie Lamarque, Marion Sabart and Delphine Latour**

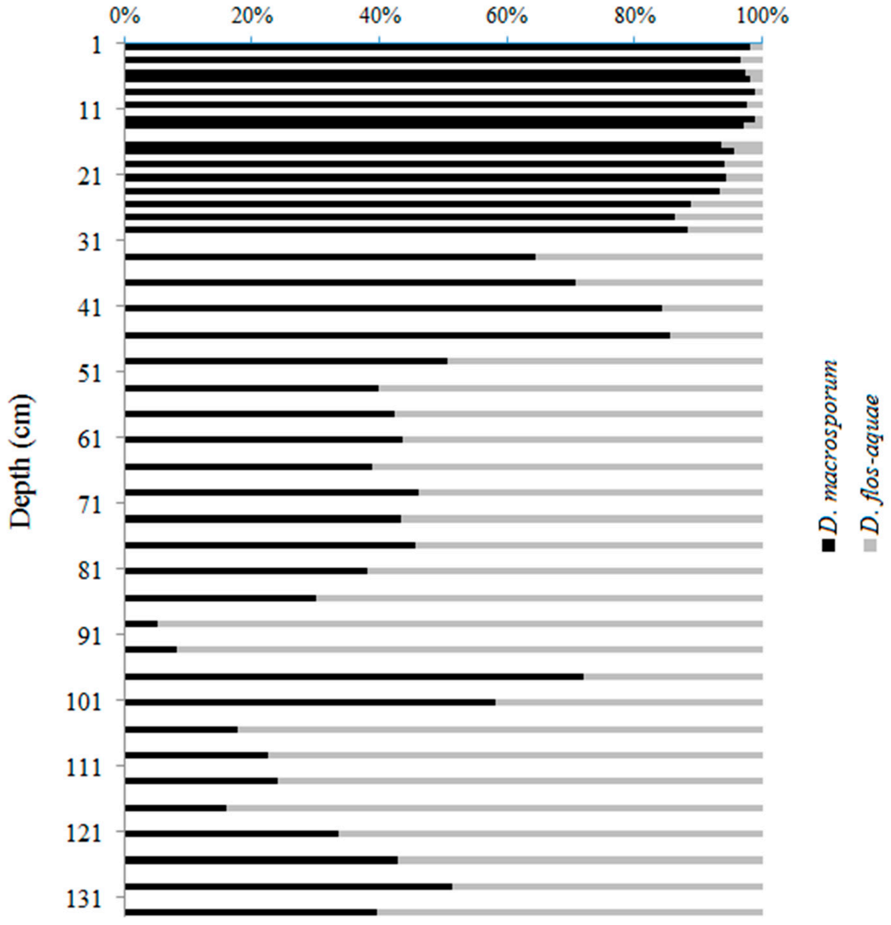


**Figure S1.** Relative percentage along the sediment core between total akinetes of *D. macrosporum* and *D. flos-aquae*.
